# Call to Action: Promoting Domestic and Global Tobacco Control by Ratifying the Framework Convention on Tobacco Control in the United States

**DOI:** 10.1371/journal.pmed.1001639

**Published:** 2014-05-06

**Authors:** Tim K. Mackey, Bryan A. Liang, John P. Pierce, Laurent Huber, Chris Bostic

**Affiliations:** 1 Department of Anesthesiology, University of California San Diego School of Medicine, San Diego, California, United States of America; 2 Global Health Policy Institute, San Diego, California, United States of America; 3 Joint Masters Program on Health Policy and Law, University of California, San Diego-California Western School of Law, San Diego, California, United States of America; 4 Department of Family and Preventative Medicine, University of California San Diego School of Medicine, San Diego, California, United States of America; 5 Action on Smoking and Health, Washington, DC, United States of America; 6 The Framework Convention Alliance for Tobacco Control, Washington, DC, United States of America

## Abstract

Tim K. Mackey and colleagues outline why the United States should ratify the Framework Convention on Tobacco Control (FCTC).

*Please see later in the article for the Editors' Summary*

**Summary Points**

- The United States is one of the few signatories that has not ratified the landmark World Health Organization Framework Convention on Tobacco Control (FCTC) public health treaty despite the passage of the Family Smoking Prevention and Control Act (FSPCA) that complies with the treaty.
- Tobacco control measures to regulate tobacco marketing and packaging have been vigorously challenged by the industry worldwide. In the US, constitutional challenges have led to a delay in legally mandated graphic warnings on packaging.
- In order to promote global tobacco control and protect future tobacco marketing regulation and interventions, the US should immediately ratify the FCTC using the FSPCA as implementing legislation. Ratification would establish FCTC provisions as domestic law under FSPCA and ensure that international norms and principles combating tobacco are achieved.
- By ratifying FCTC, the US would reinforce its own domestic tobacco control efforts and provide international support and leadership in the fight against the global tobacco epidemic. This is crucial as the industry continues to challenge tobacco regulation globally, and failure in US health policy could have a detrimental effect on FCTC implementation in other countries.

## Background

The Framework Convention on Tobacco Control (FCTC) is the only internationally binding public health treaty ever adopted under the World Health Organization's (WHO) constitution. Although an FCTC signatory, the US joins Cuba, Argentina, and a handful of other countries as one of the few signatories yet to ratify the treaty [1],[2]. With 176 ratifying countries and the European Union, FCTC has demonstrated global acceptance and progress on efforts to combat tobacco use in an effort to reduce the eight million tobacco-related deaths projected to occur annually by 2030 [3]–[5]. Within this landmark global health governance initiative are a number of mechanisms focused on both demand and supply reduction in response to the globalization of the tobacco epidemic. This focus includes demand-reduction provisions such as price/tax measures, as well as education and public awareness; protection from tobacco exposure; and, importantly, evidence-based regulation of tobacco contents, product disclosures, tobacco advertising and promotion, and tobacco packaging and labeling [6].

While it has not ratified FCTC, the US Congress implemented many of its elements in the 2009 Family Smoking Prevention and Control Act (FSPCA) [1],[7]. Given FSPCA passage, it would appear logical for the US to ratify the treaty based on the presence of existing compliant legislation in an effort to bolster its own domestic efforts in tobacco control [8]. This action would be similar to recent US ratification of the UN Environment Programme Minamata Convention on Mercury that also contains internationally binding health provisions, although historically the US has failed to ratify a number of other internationally binding treaty instruments [1],[3],[9].

Specifically, FSPCA expanded US Food and Drug Administration (FDA) authority to regulate tobacco products, including tobacco marketing. Indeed, the Act's requirements are consistent with FCTC Article 11 recommendations for graphic/pictorial warnings on ≥50% of principle package display areas. The purpose of these recommendations in both FCTC and FSPCA is to communicate information about health risks of tobacco, thus reducing cigarette marketing appeal and countering years of positive industry-based advertising [7],[10].

However, FSPCA implementation of health warning labeling mandates has been obstructed by tobacco industry legal challenges [1],[9]. These challenges are part of a broader vigorous strategy of litigation and initiation of international trade, intellectual property, and investment disputes aimed at undermining a host of global tobacco control measures [10],[11]. This includes challenges to tobacco packaging and warning initiatives in Uruguay (requiring 80% coverage of principle package display areas) and Australia's plain packaging legislation that has been upheld by the Australian high-court and recently implemented in that country [10]. Indeed, compared to other demand reduction policies contained in FCTC, international adoption of Article 11 packaging obligations is lagging behind, especially in low-income countries where tobacco use is increasing [10].

Central to domestic US litigation is the claim that FSPCA-mandated cigarette graphic warnings are a violation of constitutional commercial free speech rights [9],[12]. US courts have varied in their constitutional interpretation of these requirements, and earlier in 2013 the Supreme Court of the United States (SCOTUS) declined to hear the dispute, essentially leaving in place conflicting lower court rulings on the issue [11],[13]. However, in the likely event that industry-based challenges are raised against future labeling interventions mandated by FSPCA, SCOTUS may decide to hear these claims. At that point, SCOTUS may take into account international treaties such as FCTC when assessing the constitutionality of FSPCA [12],[14]. Hence, as we describe in this piece, we believe a renewed effort to ratify FCTC provides the proactive opportunity to strengthen FSPCA regulations against future industry legal claims and to promote US leadership in the fight against the global tobacco epidemic.

## FSPCA Legal Challenges

From one perspective, the US federal 6^th^ Circuit Court of Appeals rejected industry arguments and held the *concept* of requiring factual, graphic warning materials on cigarette packaging with effective communication of health risks is constitutional [13],[14]. From another, the federal DC Circuit Court of Appeals rejected the *specific* FDA labeling warnings proposed under FSPCA [14]. In the latter case, the appellate court struck down FDA graphic warnings, holding it was required to produce substantial evidence showing the proposed warnings were narrowly tailored and would directly accomplish the FDA's objective of reducing smoking [14],[15]. The court also felt that mandating a quitline (1-800-QUIT-NOW) on the packet was an inappropriate creation of a “billboard” against the company's own commercial interest [14]–[16]. Note, however, that in both cases the courts did not strike down the FSPCA as unconstitutional.

These appellate decisions have resulted in an indefinite delay of FDA labeling implementation. In response to these decisions, the FDA and tobacco manufacturers have taken separate strategic approaches in trying to address the legal stalemate. In March 2013, the FDA decided not to appeal the DC Circuit's ruling against FDA warning labels to SCOTUS after it had failed in its petition to the appellate court for a rehearing [11],[15]. Instead, the FDA stated it would begin the process of developing new warning labels in an attempt to address concerns raised by the DC Circuit [9],[15],[16].

Conversely, in response to the 6^th^ Circuit's ruling, the tobacco industry decided to directly challenge the ruling upholding FSPCA-mandated FDA tobacco warnings by filling a writ of certiorari with SCOTUS requesting a review and reversal of the appellate court's decision on grounds that the warnings violate commercial free speech protections [8],[11],[12]. This request was denied by SCOTUS in April 2013, effectively leaving in place the FDA's labeling authority, but also making the law's implementation subject to the adverse decision of the DC Circuit.

This state of legal ambiguity has sent the FDA to its status quo ante, with US warnings on tobacco packaging remaining in a small box and text only, not appearing on the principal display areas of packaging, and left unchanged for over two decades. Hence, the future of US tobacco graphic warnings now relies upon further development by the FDA, whose new warnings are surely to be challenged by the tobacco industry again on similar claims. Here, another potential conflict in interpretation between federal appellate circuit courts could very well bring the case and the FSPCA once again to SCOTUS for review, which could then either uphold or reject new FDA labeling. This continues to be a worrisome possibility, as SCOTUS' recent rulings have increasingly favored commercial free speech protection [9],[10].

## International Treaties and United States FCTC Implementation

The incorporation of international law into domestic law is complex and varies based on a sovereign state's own domestic legal system. This includes two different theories on the relationship between international treaties and domestic law: “monism”, whereby the act of ratification by a state's legislature immediately incorporates the treaty into domestic law (e.g., France), and “dualism”, which requires translation of international law into domestic law through enacting legislation or adapting existing domestic law to conform to treaty-bound obligations (e.g., United Kingdom). Generally, scholars agree that the United States operates under a “mixed” or “hybrid” system that incorporates monist and dualist principles that requires certain circumstances for international law to apply [17].

In order for a treaty to be implemented, once it is signed by the United States, it must then be ratified by two-thirds of the Senate under the Treaty Clause of the Constitution. Then, in order for it be given full effect, domestic law must be passed or already in compliance to implement the obligations of the treaty. These requirements have been reinforced in a recent SCOTUS decision (Medellín v. Texas [2008]), which held that an international treaty is not binding unless Congress has enacted implementing legislation *or* if the treaty is “self-executing” [18]. Though there are circumstances where international treaties are “self-executing” (such as friendship, commerce, and navigation treaties [12]), these status-based agreements require no action compared with FCTC, which has terms defining affirmative country obligations that require legislative action.

Hence, as an international treaty, the FCTC has largely been implemented through the FSPCA but the treaty itself has not been ratified [8],[12]. Note, however, that simply becoming a signatory without ratification may still create certain obligations under customary international law. Importantly, signatories generally have an obligation under customary international law to take no actions that would undermine the goals and terms of the treaty [10],[19]. Once an implementing statute for FCTC is passed, it becomes the law of the land, like other duly passed federal laws.

## FCTC Ratification to Reinforce FSPCA

To address the risk of future FDA warning labels and FSPCA mandates being subjected to further industry legal challenges, constitutional review, and potential adverse ruling, Congress and President Obama should act immediately to ratify FCTC. By ratifying the FCTC, and using the already enacted FSPCA as its implementing legislation, the FSPCA would effectively act to implement an internationally binding treaty, reinforce domestic tobacco control measures, and provide the international community support by obligating the United States to the agreed upon international norms and principles of combating the global tobacco epidemic.

Historically, this would place FSPCA in a stable position. SCOTUS has cited customary international law when interpreting enforcement of domestic law as first enumerated in the landmark Paquete Habana SCOTUS decision in 1900, and no treaty terms, once ratified and implemented, have ever been held unconstitutional in the US [19]–[21]. Specifically, upon Senate ratification, the FCTC in conjunction with FSPCA would effectively become “the supreme law of the land” in accordance with the Supremacy Clause of the US Constitution (Article VI, Clause 2) and would represent the highest form of law in the US legal system as reaffirmed in the SCOTUS decision Missouri v. Holland (1920) [22]. While SCOTUS arguably retains the authority to rule provisions of the FSPCA as unconstitutional, it also may use international law to inform decisions regarding provisions of the Constitution [17]. Hence, the existence of both FSPCA and FCTC as binding domestic and international law would mutually reinforce tobacco control regulations and positively inform future SCOTUS deliberations regarding constitutionality of the FSPCA.

Further, after ratification and implementation bill passage, SCOTUS may employ the “interpretive enforcement principle,” using FCTC's international standards and norms to interpret FSPCA, so as to take no actions that would undermine or violate the goals of the treaty [12]. Consequentially, a SCOTUS ruling striking down the implementing statute's requirements would therefore be unusual as well as a potential violation of existing international precepts. This would provide support to uphold new FDA graphic warnings under FSPCA, founded upon prevailing international norms.

In addition, ratifying FCTC also represents an opportunity to backstop a potential adverse holding that new labeling requirements are ruled by SCOTUS in future industry litigation as unconstitutional by ensuring that *minimum* internationally established tobacco labeling requirements remain (e.g., FCTC provisions stipulate at least a minimum of 30% cigarette package warning coverage and guidelines recommend rotating pictorial warnings that cover more than 50% of principal display areas). Also, by using FSPCA as the FCTC implementing statute, DC Circuit concerns can be addressed by directing domestic implementation to respond to court-deemed unsupported warning images (those ruled by the court as not conveying adequate health information; e.g., an image of a woman crying or a man with an “I Quit” T-shirt), and replacing them with “purely factual and uncontroversial” images that the FDA can develop and that are evidence-based [23]. This strategy effectively gives tobacco control proponents and legislators another “bite at the apple”: if SCOTUS holds original FSPCA mandates unconstitutional, the ratification statute represents another federal law requiring separate SCOTUS review. This would reinforce FSPCA-FCTC mandates, given that they are compatible [8], and also allow for flexibility in implementing necessary changes to labeling characteristics and requirements that the courts have had concerns with.

With ratification and implementation, FCTC will ensure a *minimum* set of tobacco control standards and support regulations in accordance with recommendations for FCTC implementation agreed upon by the international community. This would have global consequences as, despite domestic tobacco control efforts, the United States remains the 3^rd^-highest tobacco-using country, the 4^th^-largest producer of unmanufactured tobacco, and home to approximately 30% of the world's major tobacco companies (including the second-largest transnational tobacco company, Philip Morris-Altria Group) [24]–[26]. Most importantly, the US would further demonstrate its leadership and commitment to global tobacco control and the prevention of millions of deaths from tobacco-related diseases.

**Figure 1:**
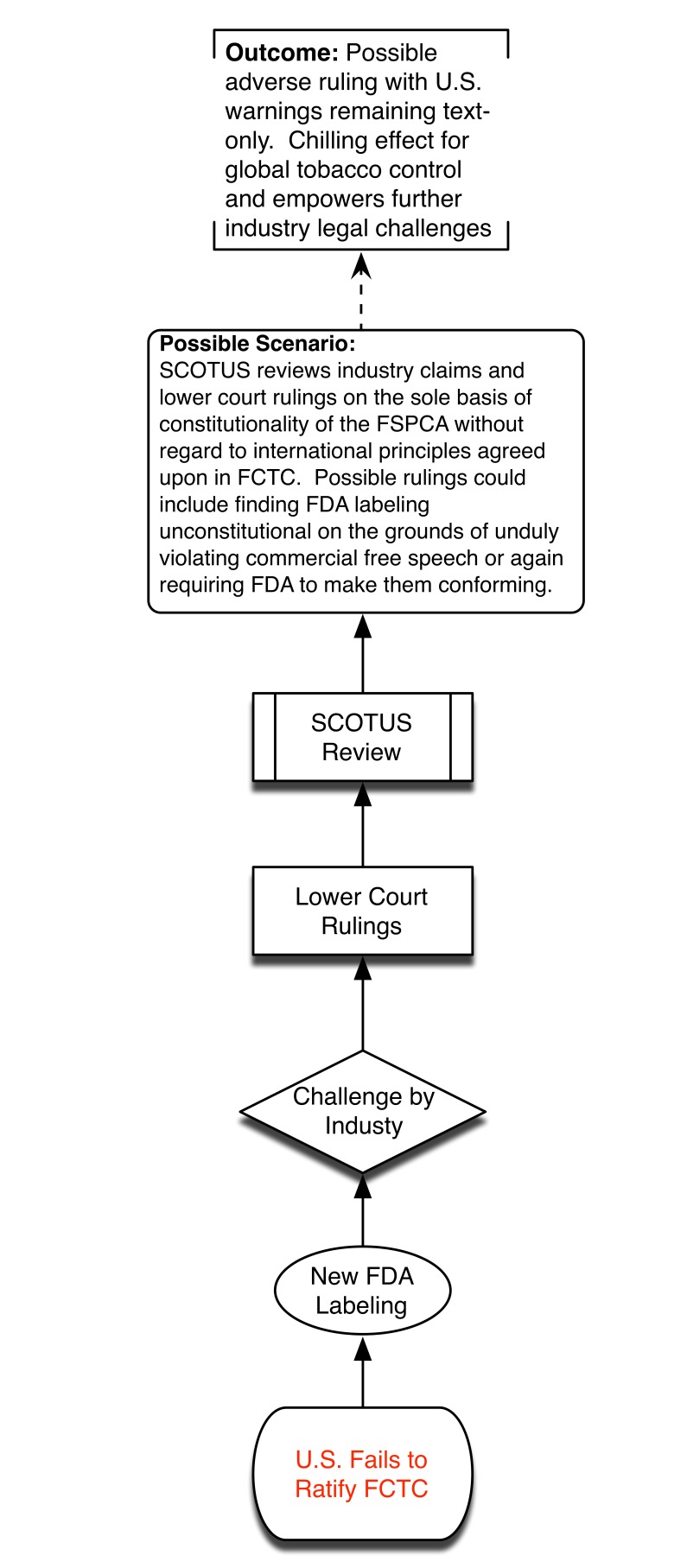
Policy scenario of US failure to ratify.

**Figure 2:**
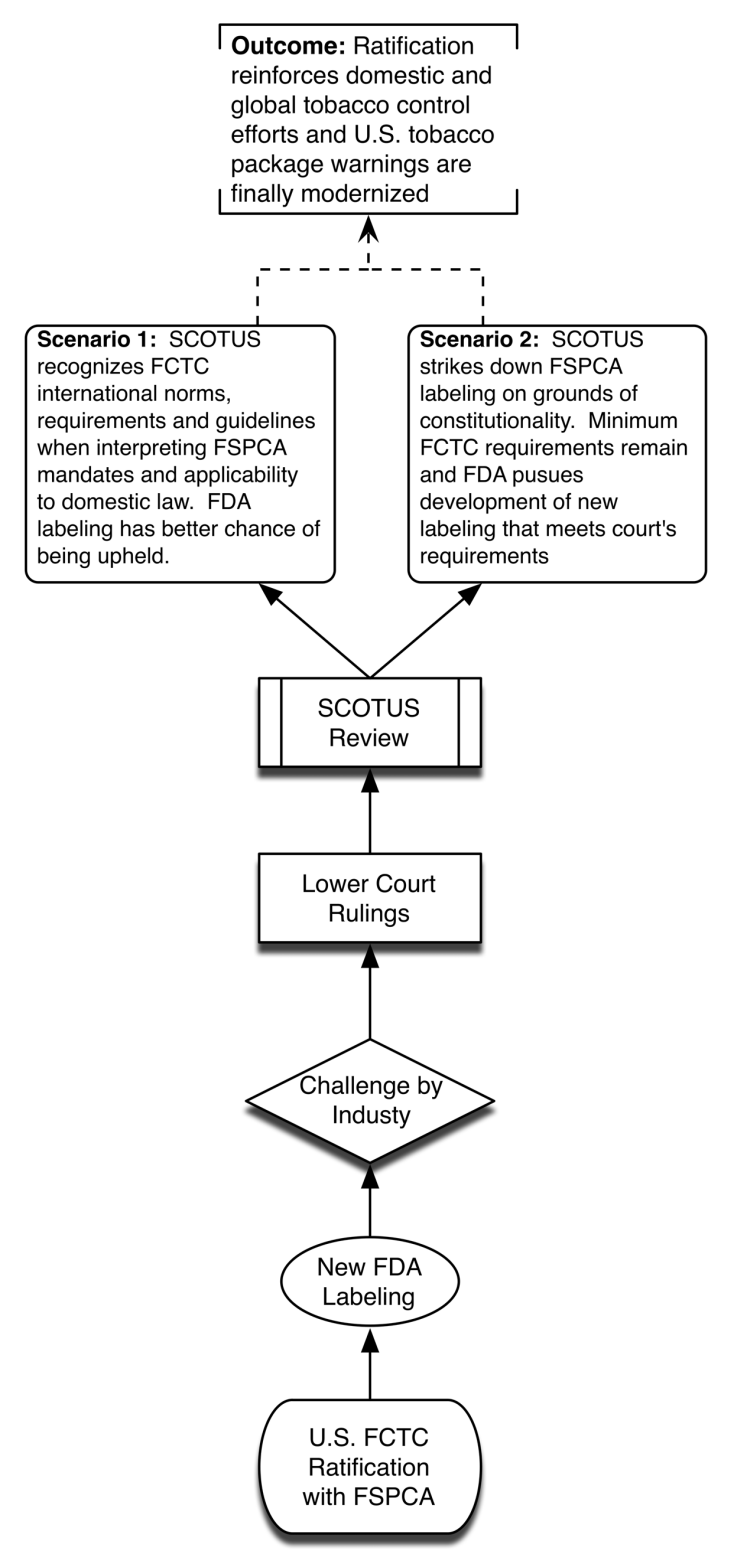
Policy scenario of successful US ratification.

Without ratification, SCOTUS may hold FSPCA and future FDA graphic warnings as unconstitutional, thus bringing years of systemic smoking control approaches back to square one. This is highly undesirable because of practical challenges of overcoming Congressional inertia to introduce de novo legislation and competing policy priorities. We provide a visual depiction of the different potential policy scenarios associated with the US failing to ratify FCTC (Figure 1) and the advantages presented by successful US ratification (Figure 2).

## Mobilizing US Support for Global Tobacco Control

Practically speaking, legislation simply assigning FSPCA to the FCTC would be direct. There is also historical support: FSPCA passed with broad legislative support (Senate passage 79-17; House 307-97) and can be used as a ratification statute by simply naming the treaty and incorporating FSPCA as the implementing statute (and the FDA as the implementing agency).

Though historically the United States has taken an obstructive stance by seeking to eliminate key FCTC provisions and pursing a “reservation” clause to allow states to opt out of certain provisions during FCTC negotiations, more than a decade since the treaty's opening for signature, the global policy environment and domestic support for international tobacco control commitment may be changing [27]. Since its opening in May 2003, the US has enacted the FSPCA, and potential US-based interference within the FCTC Conference of Parties and related implementation efforts may be minimized given that key guidelines and recommendations for FCTC articles are already in place. Ratification could also allow for more robust global tobacco control participation in key areas, such as tobacco smuggling, by allowing the United States to participate in this new FCTC protocol.

Though passage of FCTC-FSPCA ratifying legislation may face challenges, the political influence of the tobacco industry is also being challenged in several policy spaces through increased engagement by academia, philanthropic organizations, research agencies, civil society, state governments, the private sector, and even with support from members of the US Congress. These trends indicate a possible opportunity for more aggressive tobacco control policy advocacy and an opportunity to renew discussions consistent with a 2008 letter signed by then US Senator Barack Obama calling for the ratification of FCTC [8].

For example, public health stakeholders, including over 40 state attorneys general, a host of public health and medical society organizations, former New York mayor Michael Bloomberg, and close to 60 members of Congress, have called for tobacco control measures to be protected in current trade negotiations for the Trans-Pacific Partnership Agreement [28],[29]. Just recently, CVS, the United States' 2^nd^-largest drugstore chain, announced that it would end cigarette and other tobacco product sales by October 2014 as part of a repositioning of their brand in the market [30]. In addition, tobacco industry lobbying activity has experienced declines from approximately $9.2 million in 2002 to $4 million in 2012, as reported by the Center for Responsive Politics [31]. Even if Congressional opponents of domestic tobacco regulation argue that FCTC labeling requirements are unconstitutional, public opinion appears to be swaying, and adjudication of the issue would still require review by SCOTUS [32].

In turn, collective efforts by US-based stakeholders, who are already significantly contributing to global tobacco control efforts and advocating for the principles enshrined in FCTC and FSPCA, could be further enhanced by US ratification. This accomplishes shared domestic and global tobacco control policy goals. First, it avoids the outcome that FSPCA anti-smoking efforts are held unconstitutional, either in whole or as re-drafted by the FDA, in future SCOTUS rulings that the tobacco industry is sure to pursue, which would then leave the current weaker text warnings in place. Further, once ratified, even if specific FDA labeling mandates are nevertheless held unconstitutional, less restrictive treaty obligations under FCTC Article 11 can be used as a replacement since they are effectively federal law while new tobacco labeling control measures are developed.

Additionally, FCTC implementation could serve as a policy platform for finally decoupling decades of tobacco industry participation and interference in the US regulatory process by requiring the US to implement FCTC Article 5.3 [33]. This Article includes implementation guidelines requesting parties to protect public health policies from industry interference and not grant business incentives or provide preferential treatment [34]. This could also act to reinforce existing domestic orders, such as the Doggett Amendment, which prohibits US agencies from promoting the sale or export of tobacco abroad.

From a global perspective, ratification reinforces FSPCA graphic warning mandates by aligning interpretation and acknowledging prevailing international norms and practices of FCTC emphasizing the importance of tobacco control. Successful ratification could also provide important “policy-transfer” lessons on successful strategies of defending and implementing FCTC provisions in national legislation (e.g., FSPCA) for countries that experience similar constitutional restrictions on tobacco marketing regulation [35]. Hence, successful defense against US-based industry litigation and successful domestic ratification of FCTC could provide support and momentum for low- and middle-income countries seeking to implement their own tobacco control measures limiting marketing/advertising [36]. Conversely, failure could further undermine FCTC implementation and enable future global tobacco industry challenges.

## Conclusions

Current legal ambiguity has resulted in renewed efforts by the FDA to create new defensible tobacco health warnings. However, even with new warnings, a tobacco industry challenge on constitutional grounds is inevitable and could lead to a future SCOTUS hearing on the issue that poses risks for both domestic and global tobacco control efforts. This is a critical concern given the prominence of the United States in the political economy of the tobacco industry and the need for international support of state-based FCTC implementation in response to strategic and widespread industry legal challenges [10]. In response, the US, consistent with its history of progressive tobacco control policy, including the first health warnings on tobacco packages globally in 1966, should immediately and actively pursue FCTC ratification to protect and promote tobacco control measures being pursued locally and globally.

## Abbreviations

FCTC: Framework Convention on Tobacco Control
FDA: US Food and Drug Administration
FSPCA: Family Smoking Prevention and Control Act
SCOTUS: Supreme Court of the United States
